# Density-based classification in diabetic retinopathy through thickness of retinal layers from optical coherence tomography

**DOI:** 10.1038/s41598-020-72813-x

**Published:** 2020-09-28

**Authors:** Shariq Mohammed, Tingyang Li, Xing D. Chen, Elisa Warner, Anand Shankar, Maria Fernanda Abalem, Thiran Jayasundera, Thomas W. Gardner, Arvind Rao

**Affiliations:** 1 Department of Computational Medicine and Bioinformatics, University of Michigan, Ann Arbor, 48109 USA; 2 Department of Biostatistics, University of Michigan, Ann Arbor, 48109 USA; 3 Department of Ophthalmology and Visual Sciences, University of Michigan, Ann Arbor, 48105 USA; 4 Department of Radiation Oncology, University of Michigan, Ann Arbor, 48109 USA; 5 The Michigan Institute for Data Sciences, University of Michigan, Ann Arbor, 48109 USA

**Keywords:** Predictive markers, Retinal diseases

## Abstract

Diabetic retinopathy (DR) is a severe retinal disorder that can lead to vision loss, however, its underlying mechanism has not been fully understood. Previous studies have taken advantage of Optical Coherence Tomography (OCT) and shown that the thickness of individual retinal layers are affected in patients with DR. However, most studies analyzed the thickness by calculating summary statistics from retinal thickness maps of the macula region. This study aims to apply a density function-based statistical framework to the thickness data obtained through OCT, and to compare the predictive power of various retinal layers to assess the severity of DR. We used a prototype data set of 107 subjects which are comprised of 38 non-proliferative DR (NPDR), 28 without DR (NoDR), and 41 controls. Based on the thickness profiles, we constructed novel features which capture the variation in the distribution of the pixel-wise retinal layer thicknesses from OCT. We quantified the predictive power of each of the retinal layers to distinguish between all three pairwise comparisons of the severity in DR (NoDR vs NPDR, controls vs NPDR, and controls vs NoDR). When applied to this preliminary DR data set, our density-based method demonstrated better predictive results compared with simple summary statistics. Furthermore, our results indicate considerable differences in retinal layer structuring based on the severity of DR. We found that: (a) the outer plexiform layer is the most discriminative layer for classifying NoDR vs NPDR; (b) the outer plexiform, inner nuclear and ganglion cell layers are the strongest biomarkers for discriminating controls from NPDR; and (c) the inner nuclear layer distinguishes best between controls and NoDR.

## Introduction

Diabetic retinopathy (DR) is the most common complication of diabetes mellitus, affecting about one-third of all diabetic patients^1^. DR is classified into two main stages: Non-Proliferative Diabetic Retinopathy (NPDR) and Proliferative Diabetic Retinopathy (PDR). NPDR is usually diagnosed with fundus photography according to the presence of microvascular lesions (e.g. microaneurysms, retinal hemorrhage) and the absence of neovascularization^2^. NPDR can lead to impaired vision due to the presence of macular edema. When it proceeds to PDR, which is characterized by proliferation of new blood vessels in the retina, patients may experience complete vision loss due to vitreous hemorrhage and/or tractional retinal detachment. Since the introduction of optical coherence tomography (OCT) in 1991^3^, it has become a new type of biomarker for the diagnosis of DR. OCT instruments use low coherence interferometry to generate cross-sectional 3D structural imaging of the retina. When it is coupled with modern image segmentation algorithms, OCT allows accurate measurements of the thickness of individual retinal layers. Seeing the potential that such data can provide critical information to help understand the underlying mechanism of DR and discover new treatment targets, many researchers have tried to unveil the association between the thickness of retinal layers and DR progression. According to previous studies^2,4,5^, thickness changes were observed in different retinal layers and in different regions in the macula of diabetic and DR patients. Furthermore, it was found that OCT is able to detect early neurodegenerative changes in diabetic patients when DR is non-detectable using fundus exams^6^. This implies that OCT data is of great value to the early detection and better understanding of DR.

OCT-derived imaging data is a rich resource providing high-resolution pixel-level insight to visualize the variations in thickness of various retinal layers. However, when comparing the OCT of patients with diabetes but no DR (NoDR) and NPDR groups, most studies describe the thickness maps with summary statistics (e.g. average thickness across the macular region) to represent the entire retinal layer^2,4,5^, thus disregarding potentially rich information pertaining to the variation in thickness across the layers. Such summary statistics have some utility in understanding the retinal layers; however, they do not capture entire information in the distribution of the pixel-wise intensities. Consequently, sensitive small-scale changes cannot be detected through these summary features, and information on the distribution of thickness is unavoidably lost. To address this issue, instead of using summary features, we leveraged information in the entire histogram (or smoothed density profile) corresponding to pixels from the retinal layer thickness maps. This has shown to have utility in applications such as brain cancer, where intensities of the tumor region from magnetic resonance imaging were utilized to develop efficient density-based clustering approaches^7,8^. Using densities potentially aids in improving the predictive power of the classification in addition to permitting use of the entire data, not just limited to summary features.

In this paper, we present a new approach for the statistical analysis of the heterogeneity in the thickness of retinal layers. For each patient we generate a density profile corresponding to pixel values within the Early Treatment Diabetic Retinopathy Study (ETDRS) grid^9^, and these densities act as our data objects. Since the density profiles belong to the space of probability density functions (PDFs), we use the geometry of this space to establish comparisons between subjects based on their corresponding PDFs. For this purpose, we utilize the Riemannian-geometric framework that provides a metric to quantify similarity (or dissimilarity) between PDFs. We then computed principal component analysis (PCA) on the sample of PDFs to obtain predictors in the Euclidean space, which can be further used in downstream analysis through standard classification approaches. Therefore, instead of computing summary statistics, our method takes into account thickness value at each pixel and detects differences in the overall distribution of thickness. We assessed the predictive power of seven retinal layers and the overall thickness for the DR development through pairwise comparisons between controls, NoDR and NPDR subjects.

**Figure 1:**
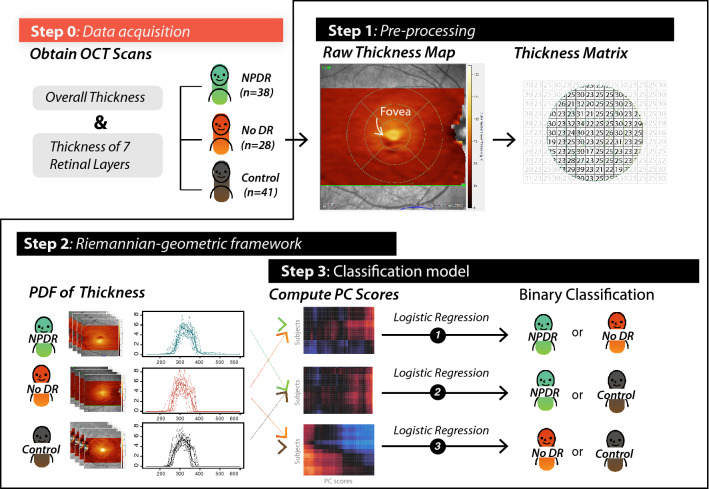
Overview of the analysis pipeline.

We describe the details of the statistical framework required for building classification models for downstream analysis in the Methods section. One of the crucial steps in our modeling approach is to identify the pixels which fall inside the ETDRS grid for a given retinal layer. Once these pixels are identified, we use them to construct the PDFs based on the pixel intensity values. These PDFs were computed as kernel density estimates using the *ksdensity* function from MATLAB with default parameters. The density estimates corresponding to each subject serve as our data objects. We then consider the Riemannian-geometric framework to build metric-based Euclidean covariates, which act as surrogates for the PDFs, through PCA and use them for distinguishing between patient groups. Hence our analytic pipeline (see Fig. 1) consists of three main components:

- Step 1. Extraction of pixel intensities from OCT images to construct the PDFs.
- Step 2. Transformation of the PDFs to allow for the comparison and modeling (with PDFs as data objects) using a comprehensive Riemannian-geometric framework.
- Step 3. Classification of the subjects using principal component scores from PCA on the space of PDFs/transformations.

This paper is organized as follows: first, we describe the data acquisition and preprocessing steps. Then we present the results for the pairwise comparisons between NPDR, NoDR and controls, and evaluate the utility of seven retinal layers and also the overall retinal thickness. We compare these predictive results with those obtained through summary statistics. Then, we include some points of discussion based on the results of our analysis. Finally, we describe in detail (a) the Reimannian-geometric framework for the space of density functions and their transformations, (b) classification model by utilizing predictors generated from the PDFs, and (c) hypothesis tests for testing differences between PDFs of two groups of subjects.

## Data

OCT scans of 107 subjects from three studies were obtained at the University of Michigan W. K. Kellogg Eye Center (Table 1). Among these subjects, 41 were healthy controls, 38 were NPDR subjects and 28 were NoDR subjects. The control group consists of subjects (age $\ge$ 18) who did not have diabetes. The NoDR group consists of subjects (age $\ge$ 18) who have been diagnosed with diabetes but no diabetic retinopathy. The NPDR group consists of patients with mild (ETDRS DR grade 20–35) or moderate (ETDRS DR grade 43–53) NPDR. Subjects with other systemic or ocular diseases that could affect vision were excluded. The OCT data sets were obtained with spectral-domain optical coherence tomography (Spectralis HR + OCT; Heidelberg Engineering, Inc., Heidelberg, Germany). Each subject had only one eye selected to be included in this study (left or right)^10^. The thickness maps of the retina (full thickness) and seven individual retinal layers (NFL: Nerve Fiber Layer; GCL: Ganglion Cell Layer; IPL: Inner Plexiform Layer; INL: Inner Nuclear Layer; OPL: Outer Plexiform Layer; ONL: Outer Nuclear Layer; RPE: Retinal Pigment Epithelium) were generated from OCT scans using the built-in segmentation algorithm of Spectralis OCT Module with default parameters. Thickness maps of each layer and the whole retina were exported manually as TIF images. Detailed information of this data set can be found in Joltikov et al.^10^.

**Table 1:** Subject characteristics.

Characteristics	Controls	Diabetes	NoDR	NPDR
**Sex**				
Female	23 (56%)	22 (34%)	12 (44%)	10 (26%)
Male	18 (44%)	43 (66%)	15 (56%)	28 (74%)
**Diabetes type**				
T1DM		19 (29%)	10 (37%)	9 (24%)
T2DM		46 (71%)	17 (63%)	29 (76%)
Age (years)	$49.1 \pm 12.9$	$54.0 \pm 13.6$	$50.5 \pm 13.8$	$56.6 \pm 13$
Diabetes Duration (years)		$15.3 \pm 11.9$	$10.5 \pm 9.9$	$18.3 \pm 12.1$
BMI (kg/m$^2$)	$25.7 \pm 3.7$	$32.2 \pm 6.8$	$31.9 \pm 7.7$	$32.4 \pm 6.3$
A1C (%)	$5.4 \pm 0.3$	$8.1 \pm 2$	$8 \pm 2$	$8.2 \pm 1.9$
Cholesterol (mg/dL)	$192.2 \pm 40.5$	$165.9 \pm 34.5$	$163.7 \pm 34.2$	$167.7 \pm 35.2$
Triglycerides (mg/dL)	$120.2 \pm 127.7$	$138.3 \pm 92.3$	$119.8 \pm 60.7$	$153 \pm 110.1$

Values are expressed as number (%) or $mean \pm SD$ Missing values were excluded to compute these summaries.

ETDRS rings of 1, 3, and 6 mm were placed on each thickness map to guide the analysis. The location of the ETDRS rings was carefully adjusted and reviewed by XDC. All left eye images were flipped horizontally before downstream analysis to make sure the thickness in each region in the ETDRS ring is comparable across images. Thickness matrices were obtained by matching pixels in the exported thickness heatmap to the closest value of the image’s color bar. Only pixels that fall inside the ETDRS ring were utilized for the analysis. Extracted thickness values are measured in units of $\upmu$m. Missing pixel values (e.g. areas where the thickness could not be measured or areas that are inside the ETDRS ring but outside of the image) are excluded from the construction of PDFs and other summary statistics.

## Results

**Figure 2:**
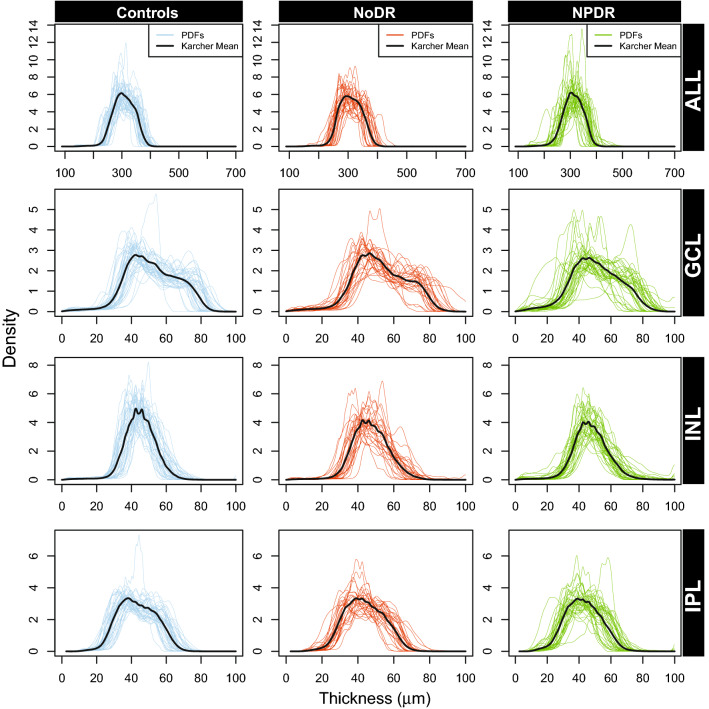
The PDFs of overall thickness (ALL), GCL, INL and IPL, corresponding to the subjects in each of the three categories: controls, NoDR and NPDR. The solid black line represents the Karcher mean of the PDFs corresponding to the subjects in each group.

**Figure 3:**
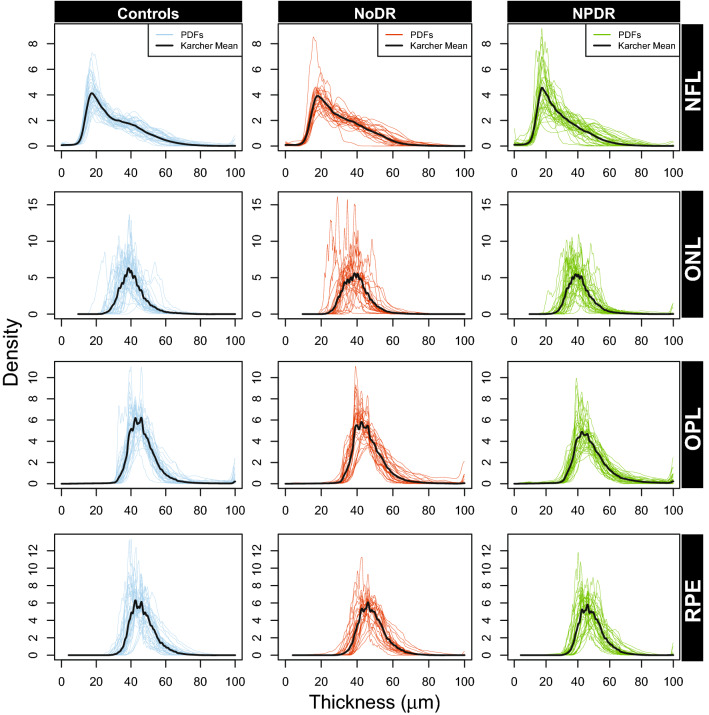
The PDFs of NFL, ONL, OPL and RPE, corresponding to the subjects in each of the three categories: controls, NoDR and NPDR. The solid black line represents the Karcher mean of the PDFs corresponding to the subjects in each group.

Our data consists of thickness of seven retinal layers as well as the overall retinal thickness. In total, 107 subjects (38 NPDR, 28 NoDR, and 41 controls) were represented in the study. Classification models were built as described in the Methods section, for the pairwise comparisons of these categories. To do this, PDFs corresponding to each of these subjects for all retinal layers were constructed separately. In Figs. 2 and 3, we showed the PDFs for each of the three patient categories corresponding to the complete thickness map and the seven retinal layers. In each of these figures, along with the subject-wise PDFs, we also plotted the Karcher mean (solid line), which is the average PDF of the subjects in each category (details in Methods section). Each of these layers were considered separately to assess their utility in the pairwise classification problem.

For each retinal layer and each pairwise comparison, we obtained the principal component scores *X* derived from the space of PDFs as described in the Methods section. Classification results from our density-based method for all three pairwise comparisons across seven retinal layers as well as overall thickness (denoted as ALL) are presented in Table 2. A leave-one-out cross-validation approach is employed for prediction. We compute the area under the receiver operating characteristic curve (AUC) and its 98.3% confidence interval (CI) which is Bonferroni corrected for multiple comparisons (to obtain a 95% overall confidence level). These are Wald-type CIs and are computed using the DeLong’s variance estimator^11^ using the pROC^12^ package in R. We also report the sensitivity, specificity and the Brier score^13^.

**Table 2:** Results of the predictive performance using density-based features as predictors for pairwise comparisons among controls, NoDR and NPDR based on seven retinal layers and overall retinal thickness.

Comparison	Layer	AUC	CI for AUC	Sensitivity	Specificity	Brier score
NoDR vs NPDR	ALL	0.514	0.338-0.691	0.579	0.571	0.351
	GCL	0.609	0.440–0.778	0.500	0.750	0.354
	INL	0.498	0.322–0.674	0.579	0.643	0.348
	IPL	0.468	0.294–0.642	0.579	0.500	0.356
	NFL	0.488	0.313–0.662	0.553	0.607	0.351
	ONL	0.621	0.451–0.791	0.658	0.607	0.355
	**OPL**	**0.679**	**0.513–0.844**	**0.632**	**0.750**	**0.294**
	RPE	0.611	0.440–0.782	0.447	0.786	0.346
Controls vs NPDR	ALL	0.488	0.328–0.648	0.632	0.512	0.377
	**GCL**	**0.703**	**0.560–0.847**	**0.763**	**0.683**	**0.319**
	**INL**	**0.799**	**0.682–0.916**	**0.711**	**0.732**	**0.276**
	IPL	0.634	0.482–0.785	0.500	0.780	0.293
	NFL	0.577	0.412–0.742	0.553	0.756	0.331
	ONL	0.493	0.329–0.657	0.474	0.634	0.355
	**OPL**	**0.802**	**0.680–0.924**	**0.789**	**0.707**	**0.224**
	RPE	0.610	0.455–0.766	0.605	0.610	0.342
Controls vs NoDR	ALL	0.646	0.485-0.808	0.821	0.463	0.394
	GCL	0.529	0.359–0.698	0.536	0.561	0.368
	**INL**	**0.703**	**0.543–0.863**	**0.643**	**0.756**	**0.285**
	IPL	0.629	0.462–0.796	0.607	0.561	0.345
	NFL	0.606	0.439–0.773	0.679	0.561	0.314
	ONL	0.535	0.361–0.709	0.607	0.561	0.368
	OPL	0.612	0.440–0.785	0.643	0.561	0.311
	RPE	0.586	0.415–0.757	0.607	0.561	0.334

ALL stands for the overall thickness. The layers which are statistically significant in demonstrating discriminatory power for each pairwise comparison (lower bound for CI > 0.5) are shown in bold font.

In the comparison between NoDR and NPDR, the OPL was identified as the best discriminator, with an AUC of 0.679 with a $98.3\%$ CI as 0.513–0.844. For controls vs NPDR, the GCL, INL and OPL were identified as statistically significant discriminators, with AUCs and the $98.3\%$ CIs of 0.703 (0.560–0.847), 0.799 (0.682–0.916), and 0.802 (0.680–0.924), respectively. The INL shows higher utility in distinguishing between controls and NoDR with an AUC of 0.703 and $98.3\%$ CI as (0.543–0.863). The $98.3\%$ CIs for AUC for all the other layers and pairwise comparisons included 0.5. Prediction results derived from simple summary statistics (five-number summary: mean, minimum, maximum, first and third quartiles) are presented in Table 3. For the comparison between controls vs NoDR, the INL had an AUC and a $98.3\%$ CI as 0.673 and (0.504–0.843). To distinguish between controls vs NPDR, the INL and OPL had an AUC and $98.3\%$ CI as 0.793 (0.674–0.912) and 0.743 (0.602–0.884), respectively. The NFL was the best in discriminating between NoDR and NPDR with the AUC and CI as 0.668 (0.508–0.828), whereas the OPL was the next best with an AUC of 0.615. The $98.3\%$ CIs for AUC for all the other layers and pairwise comparisons included 0.5. The results from Tables 2 and 3 indicate that the predictive models using the density-based features outperform the models with summary statistics in terms of the AUC and CI for the layers with reasonable discriminative power (lower bound for CI> 0.5).

**Table 3:** Results of the predictive performance using summary statistics (five-number summary) as predictors for pairwise comparisons among controls, NoDR and NPDR based on seven retinal layers and overall retinal thickness.

Comparison	Layer	AUC	CI for AUC	Sensitivity	Specificity	Brier Score
NoDR vs NPDR	ALL	0.594	0.424–0.764	0.553	0.75	0.283
	GCL	0.554	0.379–0.728	0.553	0.536	0.26
	INL	0.61	0.436–0.784	0.5	0.786	0.244
	IPL	0.615	0.437–0.792	0.684	0.643	0.255
	**NFL**	**0.668**	**0.508–0.828**	**0.579**	**0.714**	**0.289**
	ONL	0.579	0.409–0.749	0.711	0.464	0.256
	OPL	0.615	0.444–0.785	0.711	0.571	0.25
	RPE	0.573	0.398–0.748	0.684	0.536	0.258
Controls vs NPDR	ALL	0.549	0.389–0.710	0.447	0.78	0.281
	GCL	0.613	0.460–0.766	0.605	0.61	0.256
	**INL**	**0.793**	**0.674–0.912**	**0.763**	**0.683**	**0.191**
	IPL	0.586	0.428–0.744	0.658	0.585	0.258
	NFL	0.647	0.494–0.800	0.711	0.61	0.241
	ONL	0.615	0.459–0.770	0.579	0.634	0.244
	**OPL**	**0.743**	**0.602–0.884**	**0.684**	**0.805**	**0.204**
	RPE	0.608	0.453–0.764	0.605	0.707	0.253
Controls vs NoDR	ALL	0.525	0.351–0.700	0.571	0.561	0.265
	GCL	0.65	0.488–0.812	0.679	0.61	0.286
	**INL**	**0.673**	**0.504–0.843**	**0.607**	**0.634**	**0.238**
	IPL	0.557	0.384–0.729	0.571	0.561	0.255
	NFL	0.641	0.481–0.802	0.607	0.634	0.238
	ONL	0.601	0.431–0.771	0.571	0.659	0.248
	OPL	0.642	0.474–0.810	0.571	0.707	0.239
	RPE	0.504	0.329–0.680	0.571	0.537	0.261

ALL stands for the overall thickness. The layers which are statistically significant in demonstrating discriminatory power for each pairwise comparison (lower bound for CI > 0.5) are shown in bold font.

For the comparison between NoDR vs NPDR, NFL performs best with summary statistics as predictors whereas OPL is most predictive with the density-based features. Note that although their AUCs are the highest (compared to other layers) for NoDR vs NPDR, the lower bounds of the associated CIs are close to 0.5. Hence, these results need to be interpreted with caution unlike other comparisons (e.g. INL in controls vs NPDR), where the indication of performance is stronger both in terms of AUCs and their CIs. Additionally, the evident differences (wiggly peaks) between average PDFs of NoDR and NPDR in OPL (see Fig. 3), are better captured by density-based features compared to summary statistics that fail to capture such nuances in the distribution of pixel intensity values. Similar arguments hold for the comparison between controls vs NPDR in GCL (see Fig. 2). However, the average PDFs of NFL in NoDR and NPDR (see Fig. 3) do not exhibit any evident visual differences. In this case, the PDF-based approach might not be adding additional insight compared to the summary statistics. All other discriminative layers show concordant performance in terms of AUCs with the density-based features performing better for the best predictive layers. Therefore, the ability of our functional data approach to capture small-scale changes in the PDFs of such high-resolution OCT images makes it a useful complement to existing approaches using summary statistics.

**Figure 4:**
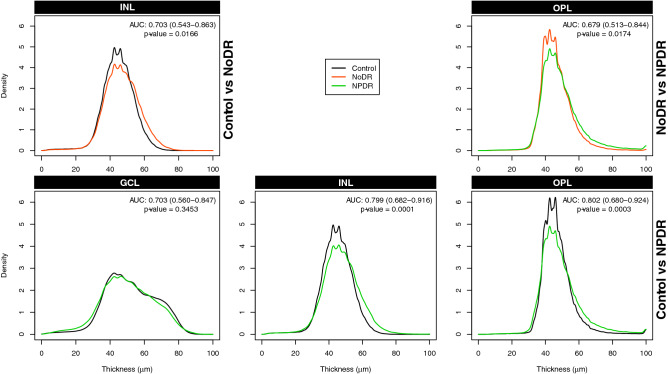
Karcher means of densities corresponding to the retinal layers with better distinction abilities for pair-wise comparisons from Table 2.

In Fig. 4, we show the pairwise comparison of Karcher means between groups for those layers which show better predictive performance in Table 2. This visual representation of the mean PDFs shows the differences in thickness profiles between the two groups based on sample averages of the PDFs. For example, the INL is most predictive for the comparison between NoDR and controls with an AUC of 0.703 and a $98.3\%$ CI as (0.543–0.863). The plot in the top-left panel in Fig. 4 displays a higher peak for controls at $40 \; \upmu$m thickness; however, NoDR has slightly higher right tail. Specifically, from the mean PDFs we see that 29% of the ETDRS grid region has more than $50\mu m$ thickness in controls compared to 39% in NoDR. These percentages were computed using the quantiles of the Karcher means. The top-left panel in Fig. 4 indicates that the INL has slightly higher thickness in the NoDR compared to the controls. To further investigate this, we perform permutation-based hypothesis tests using the sample PDFs for each group of subjects. We test the hypothesis that the average PDFs of the groups are similar to each other or not. The details of the permutation test are given in the Methods section. In Fig. 4 and Table 4 we report the p-value from this test to quantify the statistical differences between the two groups of PDFs. The p-values are Bonferroni adjusted for multiple comparisons. For the INL in the comparison between controls and NoDR, the p-value for the permutation test is 0.0166 indicating statistically significant differences between the average PDFs of the two groups.

We perform a similar analysis for the other two pairwise comparisons. For the comparison between controls and NPDR, the GCL, INL and OPL show better predictive performance with the AUC and the $98.3\%$ CI as 0.703 (0.560–0.847), 0.799 (0.682–0.916) and 0.802 (0.680–0.924), respectively. The p-values for the permutation tests for these three layers are 0.3453, 0.0001 and 0.0003, respectively. This indicates significant differences between the average PDFs of the thickness profiles of OPL and INL. However, the test does not reveal statistically significant differences in GCL between the controls and NPDR, which is consistent from the similar average PDFs for controls and NPDR in GCL (bottom-left panel in Figure 4), and its lower AUC compared to INL or OPL. From the mean PDFs for INL we see that 29% of the ETDRS grid region has a thickness of more than $50\;\upmu$m in controls compared to 40% in NPDR. Similarly, a thickness of more than $50\;\upmu$m is observed in 30% of the ETDRS grid region of OPL in controls compared to 38% in NPDR. Specifically, from Fig. 4 we see that the thickness of the INL and OPL is slightly higher in the NPDR compared to the controls. This is also indicated due to the higher peak for controls near $40\;\upmu$m thickness and a heavier right tail for NPDR. For the comparison between NoDR and NPDR, the OPL shows reasonable predictive performance with the AUC and the $98.3\%$ CI as 0.679 (0.513–0.844). The p-value for the permutation test is 0.0174, which identifies significant differences between the average PDFs between NoDR and NPDR, indicating evidence of slightly higher thickness of OPL in the NPDR. Specifically, 30% of the ETDRS grid region in OPL has a thickness of more than $50\mu m$ in controls compared to 38% in NPDR. The p-values for all the other layers and pairwise comparisons are provided in Table 4. Note that these p-values are Bonferroni corrected for the three pairwise comparisons.

**Table 4:** The p-values from the permutation-based hypothesis tests to test differences between the average PDFs of the subjects in each pairwise comparison.

Pairwise comparison	ALL	GCL	INL	IPL	NFL	ONL	OPL	RPE
NoDR vs NPDR	1.0000	0.3453	0.4806	1.0000	0.4436	0.3729	**0.0174**	0.8941
Control vs NPDR	0.9162	0.3453	**0.0001**	0.7062	0.3117	0.7252	**0.0003**	0.1689
Control vs NoDR	1.0000	0.5713	**0.0166**	1.0000	0.4436	0.4614	0.2768	0.2422

Statistically significant differences are shown in bold font.

**Figure 5:**
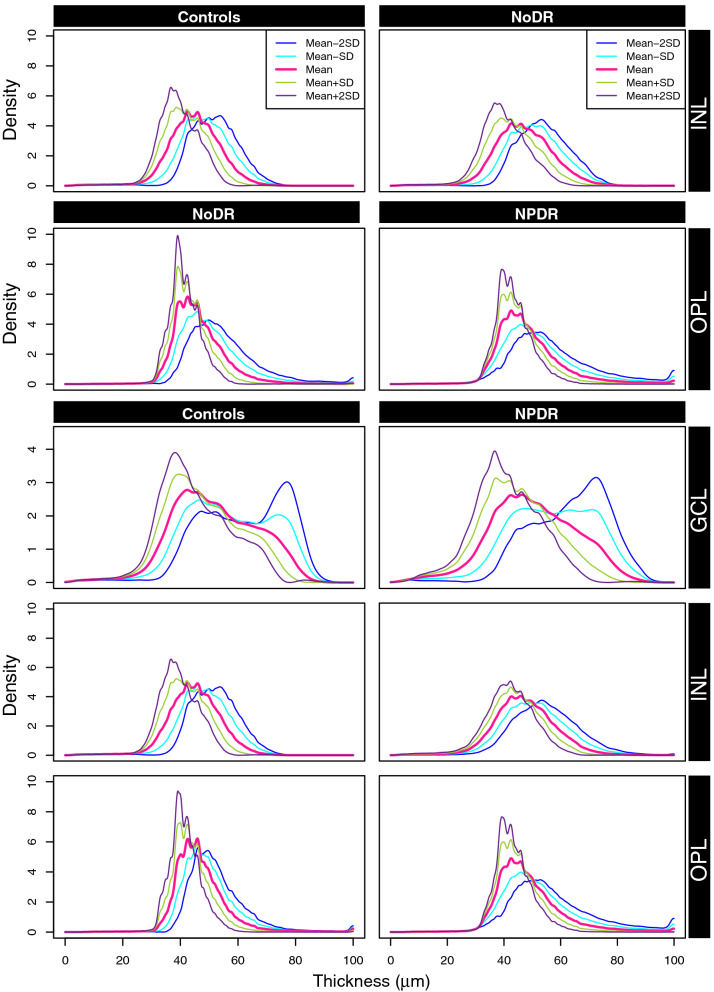
First principal direction of variability in a specific layer from a given group of subjects. In each case we present the path sampled with − 2, − 1, 0, + 1, + 2 standard deviations around the mean along the first principal component direction.

We further investigate these differences in thickness of the layers through the variability in the subjects within each group. Figure 5 shows the path sampled with − 2, − 1, 0, + 1, + 2 standard deviations around the mean along the first principal component direction transformed back to the space of PDFs. In the comparison between controls and NoDR for the INL, the sample path of PDFs in the first row of Fig. 5 indicates that these PDFs have slightly higher peaks at lower thickness values in controls compared to NoDR. Similar results can be observed for (a) the comparison between NoDR and NPDR in the OPL (second row in Fig. 5), and (b) the comparison between controls and NPDR in the INL and OPL (fourth and fifth rows in Fig. 5). However, the differences in GCL between the controls and NPDR (third row in Fig. 5) are not apparent, which is in agreement with our earlier result in terms of the smaller AUC for GCL. From these results, we see that we achieve better classification of subjects when each of the retinal layers are considered separately instead of solely looking at the complete retinal thickness. Each of these retinal layers are either thickened or thinned for a group of subjects and some of them offer better distinction ability for specific pairwise comparison between groups.

## Discussion

We evaluated the utility of seven retinal layers and the overall thickness for the pairwise comparison between different severity levels of DR. Raw pixel intensities were transformed into density functions under a Riemannian-geometric framework and then principal component analysis was applied to generate sample specific features. Our density-based method was applied to a prototype data set and showed considerable improvement in predictive power when using individual layers as predictors instead of the total retinal thickness. According to our observations, OPL demonstrates the best performance in classifying NPDR versus NoDR. Similarly, for the classification of controls versus NPDR we see that OPL, INL and GCL provide the best performance. INL is able to distinguish between NoDR and controls. These findings are consistent with results from previous studies that demonstrated the disruption of inner retinal layers early in the course of diabetic retinopathy^14–17^. Hence, our analysis has identified potential biomarkers in distinguishing between each category of the severity of diabetic retinopathy and should be further investigated.

The postmortem human retina from donors with diabetes is found to have increased number of cell deaths in retinal neural cells even in areas away from the microvascular lesions^14^. Apoptosis of retinal neural cells was observed in diabetic rats induced by streptozotocin^14^. Consequently, their retina had 10% loss of surviving ganglion cells and significant thinning of IPL and INL after 7.5 months of streptozotocin. In a separate experiment, streptozotocin diabetic rats killed after 12 months also had thinner GCL and INL, though changes in INL were more remarkable^15^. Interestingly, several studies have reported retinal NFL defects as an early sign of DR^18–20^. Significant loss in retinal NFL was observed in patients with type 1 diabetes without retinopathy using scanning laser polarimetry^19^. Other studies^18,20^ also showed that the retinal NFL becomes thinner and the nerve fiber defects in the retina increase as retinopathy progresses. However, our analysis suggests that changes in the INL associated with diabetes have higher differentiating capabilities than the early loss of NFL. In our study, we showed that the change in INL was the best indicator to differentiate NoDR from controls. This is validated from our results due to the discriminative capability and the p-values corresponding to INL in the comparison between controls and NPDR. Also, disorganization of retinal inner layers (DRILs) is associated with inner retinal thinning in patients with NoDR^10,21^. When comparing between NPDR and NoDR or controls, inner nuclear, outer plexiform, and ganglion cell layers were better predictors. Hence, future studies should investigate the mechanisms of these pathological changes, which may provide information about the progression of this disease and identify new therapeutic target for future treatments.

Our method has multiple advantages when compared to models which use summary statistics from the images as predictors. Our approach is based on a principled way of analyzing the heterogeneity in the thickness values from the high-resolution OCT images. We use the entire distribution of the pixel-wise thickness and capture the complete information presented. In addition, our method is invariant to scaling and shifting of thickness values and can be applied to thickness measured by different OCT instruments. Lastly, using PDF-based features (i.e. the principal component scores) instead of scalar summaries (i.e. the average thickness) to represent each sample allows the construction of more sophisticated machine learning models. By constructing densities which best model the heterogeneity in our sample data, we create more illustrative models of the sample distribution, which enable richer contextual inferences, such as our developments on layer variation mentioned above. Exploring the space of densities provides better insight into the heterogeneity of the thickness distributions. Consequently, variability in these density functions is effectively captured through the principal components although the mean densities of certain groups visually appear to be similar. As part of our future works we plan to further investigate these findings in terms of the utility of the layers with larger sample sizes to establish comparison with controls matched for specific clinical characteristics. Additionally, a larger sample size for each pairwise comparison with a reasonable way to split the data into training and validation sets that captures the complete underlying variation would facilitate meaningful validation.

With the recent advancements in the field of data analysis and specifically its applications to imaging data, many machine learning approaches have been deployed to learn from the data. However, such algorithms usually require huge amounts of data and computational tools to efficiently train the model. In this specific application to diabetic retinopathy, obtaining large amounts of OCT data is challenging due to the associated costs. In such situations, making meaningful inferences from the data available is only feasible by employing model-based analytic approaches. With 38 NPDR, 28 NoDR and 41 controls, the sample size is a limitation of our OCT data to perform pairwise comparisons. However, our density-based modeling approach provides an efficient and innovative alternative by maximizing the utility of the information in the data. Further investigation involving more samples will facilitate comparison with matched controls.

In summary, this study showed that, the density of thickness values measured by the OCT instrument is able to capture the information that cannot be captured by the descriptive statistics. It can serve as an important biomarker of NPDR to aid the research and diagnosis of DR. Our proposed proof-of-concept study could be applied to larger cohorts of OCT data and other ophthalmology imaging data to understand the retinal structuring in DR which could arise due to subtle and small-scale changes in the thickness of the various layers.

## Methods

### Space of probability densities and their transformations

The PDFs computed based on the pixel values inside the ETDRS grid, lie on a non-linear functional space. We exploit the differential geometry of this space, however, we restrict ourselves to the case of univariate densities on [0, 1]. For this purpose, we normalize the PDFs so that they belong to the Banach manifold ${\mathscr {F}}= \{f:[0,1] \rightarrow \mathbb {R}_{\ge 0} | \int _0^1 f(x) dx = 1 \}$. Note that ${\mathscr {F}}$ has a boundary which contains all PDFs for which the normalized pixel values become 0 anywhere on the domain. For any point *f* a tangent space can be defined as $T_f({\mathscr {F}}) = \{ \delta f: [0,1] \rightarrow \mathbb {R} | \int _0^1 f(x) \delta f(x) dx = 0 \}$, which is a vector space of all possible perturbations of *f*. A Reimannian metric on ${\mathscr {F}}$ can be used to compute geodesic distances between two PDFs and also the summary statistics of a sample of PDFs. The non-parametric Fisher-Rao metric (FR metric)^22–24^ for any two tangent vectors $\delta f_1, \delta f_2 \in T_f({\mathscr {F}})$ is defined as $\langle \langle \delta f_1, \delta f_2 \rangle \rangle _f = \int _0^1 \delta f_1(x) \delta f_2(x)\frac{1}{f(x)} dx$. This FR metric has nice mathematical properties such as being invariant to reparameterizations of densities^25^. However, one of the drawbacks of this metric is the challenge it presents in computing the geodesic paths and distances. This challenge arises as the metric changes from point to point on ${\mathscr {F}}$.

#### Square-root transformations of the PDFs

Since our goal is to build classification models, we want to work with data objects in a suitable representation of the space ${\mathscr {F}}$, where the geometry is not as complex as in the Banach manifold ${\mathscr {F}}$. Specifically, we work with transformations on the PDFs such that the complex non-linear space changes to a much simpler space where the computation is feasible. One such convenient choice of representation for PDFs, is the square-root transformation (SRT), $h = +\sqrt{f}$^26^, since it allows to measure the distance between any two points in ${\mathscr {F}}$ as a standard $\mathbb {L}^2$ Reimannian metric^27^. We omit the ‘+’ sign hereafter for notational convenience. The inverse mapping is unique and is simply given by $f = h^2$, and hence the space of the SRTs corresponding to ${\mathscr {F}}$ is given by ${\mathscr {H}}= \{h:[0,1] \rightarrow \mathbb {R}_{\ge 0} | \int _0^1 h^2(x) dx = 1 \}$. Here ${\mathscr {H}}$ represents the positive orthant of the unit Hilbert sphere^28^. The tangent space at any point $h \in {\mathscr {H}}$ is defined as $T_h({\mathscr {H}}) = \{\delta h :[0,1] \rightarrow \mathbb {R} | \int _0^1 h(x) \delta h(x) dx = 0 \}$. The FR metric can now be defined using the geometry of the space of SRTs. The geodesic distance between two densities $f_1,f_2 \in {\mathscr {F}}$, represented by their SRTs $h_1,h_2 \in {\mathscr {H}}$, is defined as the shortest arc connecting them on ${\mathscr {H}}$, that is, $d(h_1,h_2) = \theta := cos^{-1} \big ( \int _0^1 h_1(x) h_2(x) dx \big )$. This is also the standard $\mathbb {L}^2$ distance between $h_1,h_2 \in {\mathscr {H}}$. Geodesic distance between two PDFs can now be computed in an efficient manner as the $\mathbb {L}^2$ Riemannian geometry of the unit sphere is well known.

#### Karcher mean for a sample of PDFs

The geometry of ${\mathscr {H}}$ can also be used to define an average (mean) PDF, which is a representative PDF of the pixel intensity values for a sample of subjects. This average PDF allows us to visualize and summarize the PDFs from the sample. Let $f_i$ denote the PDF corresponding to the pixel values inside the ETDRS grid for subject *i* for all $i=1,\ldots ,n$ and $h_1,\ldots ,h_n$ be their corresponding SRTs. A generalized version of the mean on a metric space that can be used to compute the average density is called the Karcher mean^29^. Specifically, as the unique inverse transformation^27^ of the SRT is given by $f = h^2$, the sample average of PDFs $f_1,\ldots ,f_n$ can be computed as $\bar{f} = \bar{h}^2$, where $\bar{h}$ is the sample average on the space of SRTs. The sample Karcher mean $\bar{h}$ on ${\mathscr {H}}$ is the minimizer of the Karcher variance $\rho (\bar{h}) = \sum _{i=1}^n d(\bar{h},h_i)^2_{\mathbb {L}^2}$, that is, $\bar{h} = \text {argmin}_{h \in {\mathscr {H}}} ~\rho (\bar{h})$. Gradient-based iterative approaches are utilized to compute the Karcher mean on $\Psi$^30,31^. Note that the Karcher mean of the sample SRTs is an intrinsic average that is computed directly on $\Psi$ (or equivalently ${\mathscr {F}}$), hence we have a mean which is an actual PDF.

The computations require important tools from differential geometry called the exponential and inverse-exponential maps. For $h \in {\mathscr {H}}$ and $\delta h \in T_h({\mathscr {H}})$, the exponential map at *h*, $\exp : T_h({\mathscr {H}}) \rightarrow {\mathscr {H}}$ is defined as $\exp _h(\delta h) = cos(||\delta h||)h + sin(||\delta h||)\delta h/||\delta h||$, where $||\delta h||^2 = \int _0^1 \big (\delta h(x)\big )^2 dx$. For any $h_1, h_2 \in {\mathscr {H}}$, the inverse-exponential map is denoted by $\exp ^{-1}_{h_1}: {\mathscr {H}}\rightarrow T_{h_1}({\mathscr {H}})$ and is defined as $\exp ^{-1}_{h_1} (h_2) = \theta [h_2 - cos(\theta )h_1]/sin(\theta )$.

#### Principal component analysis

Under the standard settings, visualizing the space of PDFs intuitively and understanding the variability in a sample of PDFs is difficult. PCA is an effective approach to explore the variability in the PDFs through their primary modes of variation. We can linearize the data representation space via the tangent space at the mean, $T_{\bar{h}}({\mathscr {H}})$, and compute Euclidean coordinates in this space conveniently due to its Riemannian structure. Note that the tangent space is a vector (Euclidean) space, hence PCA can be implemented as in standard problems. Algorithm 1 outlines the implementation of PCA on the tangent space at the mean of SRTs $h_1,\ldots ,h_n$ (corresponding to PDFs $f_1,\ldots ,f_n$).

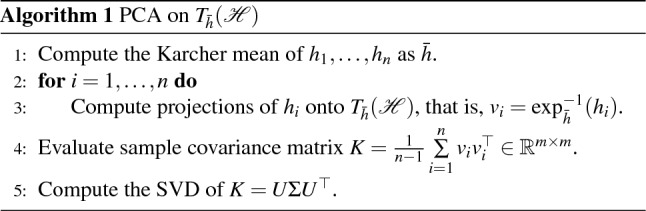



Here the orthogonal matrix *U* contains the principal components or principal directions of variability, and the diagonal matrix $\Sigma$ contains the singular values. The number of subjects is usually smaller than the dimensionality of each tangent vector, i.e., $n \ll m$. Note that the first *r* columns of *U* (denoted as $\tilde{U} \in \mathbb {R}^{m \times r}$) span the *r*-dimensional principal subspace. The choice of *r* could be made based on the cumulative amount of variance explained by the first few principal components. We can express the data using coordinates in this subspace via principal coefficients computed as $X = V\tilde{U}$, where $V^\top = [v_1~ v_2~ \ldots ~ v_n] \in \mathbb {R}^{m \times n}$. These principal coefficients *X* act as Euclidean coordinates corresponding to densities and can be used as predictors for downstream analysis.

Note that for our pairwise analysis the tangent space is considered at the Karcher mean of PDFs corresponding to only the subjects from the two categories in the pair being evaluated. The number of columns to include in X is determined as the number of principal components required to explain 99.99% of the total variation.

### Classification model

Our main goal is to build classification models to discriminate between any two categories of subjects. To address this we can consider the principal coefficients *X* as our predictors. These principal coefficients are predictors derived from the PDFs corresponding to the image of a retinal layer. Let us consider the variable $y_i \in \{0,1\}$ as the response indicating the class membership of the subject *i* for all $i=1,\ldots ,n$. For each of subject *i*, using the retinal layer map we can construct a PDF $f_i$ based on the pixel values in the ETDRS grid. Using the approach discussed earlier in this section, we can construct the corresponding principal component scores $X \in \mathbb {R}^{n \times r}$ which can further be used as covariates in the Euclidean space. Once we obtain the covariates *X* and the response ${\mathbf {y}}= (y_1,\ldots ,y_n)$, we can use standard classification algorithms to build discriminative models.

In this paper we considered logistic regression which is a generalized linear model used to model a binary categorical variable using numerical and/or categorical predictors. We assume a binomial distribution produced the outcome variable and we therefore want to model $p_i = P(y_i = 1)$, the probability that a subject belongs to the category 1 for a given set of predictors. More specifically, in the logistic regression we model the log-odds as a linear combination of the predictors as $\log \big (\frac{p_i}{1-p_i}\big ) = {\mathbf {x}}_i^\top {\beta }$, where ${\mathbf {x}}_i$ is the *i*th row in *X* and ${\beta }\in \mathbb {R}^r$. Standard estimation approaches can be used to estimate the coefficients ${\beta }$. Once we obtain the estimated coefficients $\hat{{\beta }}$, we can use them to predict the probability of class membership for a new subject. That is, for a new subject, we can estimate $\hat{p}_{new} = 1/\big (1+e^{-{\mathbf {x}}_{new}^\top \hat{{\beta }}}\big )$. We considered logistic regression, but other classification algorithms can also be used to build classification models.

### Hypothesis testing

We build a permutation-based hypothesis test to further investigate differences in the PDFs corresponding to the subjects for a given layer. That is, we want to investigate the hypothesis that the average PDFs for the groups (based on the binary response) are similar to each other or not. The similarity between these average PDFs is quantified by the geodesic distance between them. Using similar notation as before, let $y_i \in \{0,1\}$ and $f_i$ denote the binary response and the PDF for subject *i*, respectively. Let $\bar{f}^0$ and $\bar{f}^1$ denote the Karcher mean of the PDFs for the subjects corresponding to $y_i = 0$ and $y_i = 1$, respectively. We define $d_0 = d(\bar{h}^0,\bar{h}^1)$ as the distance between $\bar{f}^0$ and $\bar{f}^1$, where $\bar{h}^0$ and $\bar{h}^1$ are the SRTs corresponding to $\bar{f}^0$ and $\bar{f}^1$. This value of $d_0$ serves as our test statistic for the hypothesis test.

We create the null distribution corresponding to the test statistic by randomly permuting the response labels $y_i$ between the subjects $i=1,\ldots ,n$. Let ${\mathbf {y}}_\sigma = (y_{\sigma (1)},\ldots ,y_{\sigma (n)})$ denote a random permutation of the response labels $y_1,\ldots ,y_n$. Using the permuted response labels ${\mathbf {y}}_\sigma$ and the original PDFs $f_1,\ldots ,f_n$ we compute the group average PDFs $\bar{f}^0_\sigma$ and $\bar{f}^1_\sigma$, and the distance between these average PDFs as $d_\sigma$. We repeat this process *m* times by considering the permutations $\sigma _1,\ldots ,\sigma _m$ and obtain the distance between the group average PDFs for each permutation as $d_{\sigma _1},\ldots ,d_{\sigma _m}$, which serve as the null distribution for our test statistic $d_0$. The p-value for this permutation-based hypothesis test is computed as $\sum _{k=1}^m I(d_0 > d_{\sigma _k}) /m$, where $I(d_0 > d_{\sigma _k}) = 1$ if $d_0 > d_{\sigma _k}$, and 0 otherwise.

### Approval, accordance and informed consent

This study involving human subjects was approved by University of Michigan Institutional Review Board and conducted in accordance to the Declaration of Helsinki. All patients signed an informed consent form prior to enrollment.

## Data Availability

The data that support the findings of this study are available from University of Michigan Medical School but restrictions apply to the availability of these data, which were used under license for the current study, and so are not publicly available. Data are however available from the authors upon reasonable request and with appropriate permissions of University of Michigan Medical School.
